# To What Extent is Primate Second Molar Enamel Occlusal Morphology Shaped by the Enamel-Dentine Junction?

**DOI:** 10.1371/journal.pone.0138802

**Published:** 2015-09-25

**Authors:** Franck Guy, Vincent Lazzari, Emmanuel Gilissen, Ghislain Thiery

**Affiliations:** 1 CNRS INEE UMR 7262 – IPHEP, Institut de Paléoprimatologie et Paléontologie Humaine, Evolution et Paléoenvironnements. Université de Poitiers – Faculté des Sciences, Bât. B35 –TSA 51106, 6 rue Michel Brunet, 86073, Poitiers, Cedex 9, France; 2 Department of African Zoology, Royal Museum of Central Africa, B-3080, Tervuren, Belgium; 3 Laboratory of Histology and Neuropathology, Université Libre de Bruxelles, B-1070, Brussels, Belgium; Museo Nazionale Preistorico Etnografico 'L. Pigorini', ITALY

## Abstract

The form of two hard tissues of the mammalian tooth, dentine and enamel, is the result of a combination of the phylogenetic inheritance of dental traits and the adaptive selection of these traits during evolution. Recent decades have been significant in unveiling developmental processes controlling tooth morphogenesis, dental variation and the origination of dental novelties. The enamel-dentine junction constitutes a precursor for the morphology of the outer enamel surface through growth of the enamel cap which may go along with the addition of original features. The relative contribution of these two tooth components to morphological variation and their respective response to natural selection is a major issue in paleoanthropology. This study will determine how much enamel morphology relies on the form of the enamel-dentine junction. The outer occlusal enamel surface and the enamel-dentine junction surface of 76 primate second upper molars are represented by polygonal meshes and investigated using tridimensional topometrical analysis. Quantitative criteria (elevation, inclination, orientation, curvature and occlusal patch count) are introduced to show that the enamel-dentine junction significantly constrains the topographical properties of the outer enamel surface. Our results show a significant correlation for elevation, orientation, inclination, curvature and occlusal complexity between the outer enamel surface and the enamel dentine junction for all studied primate taxa with the exception of four modern humans for curvature (p<0.05). Moreover, we show that, for all selected topometrical parameters apart from occlusal patch count, the recorded correlations significantly decrease along with enamel thickening in our sample. While preserving tooth integrity by providing resistance to wear and fractures, the variation of enamel thickness may modify the curvature present at the occlusal enamel surface in relation to enamel-dentine junction, potentially modifying dental functionalities such as blunt versus sharp dental tools. In terms of natural selection, there is a balance between increasing tooth resistance and maintaining efficient dental tools. In this sense the enamel cap acts as a functional buffer for the molar occlusal pattern. In primates, results suggest a primary emergence of dental novelties on the enamel-dentine junction and a secondary transposition of these novelties with no or minor modifications of dental functionalities by the enamel cap. Whereas enamel crenations have been reported by previous studies, our analysis do not support the presence of enamel tubercles without dentine relief nuclei. As is, the enamel cap is, at most, a secondary source of morphological novelty.

## Introduction

For nearly 200 years evolutionary biologists have commonly used tooth morphology as a basis for tracing mammalian evolutionary history [1]. Extant dental diversity arose from successive mammalian radiations and results in part from specific dietary adaptations. Recent decades have been significant in unveiling developmental processes controlling teeth morphogenesis, dental variation and the origination of dental novelties [2–5]. Tooth shape is determined by the folding and growth of the interface between the oral epithelium and the mesenchyme from which originate the enamel-forming ameloblasts and the dentine-forming odontoblasts [6]. The subsequent enamel-dentine junction (EDJ) constitutes a precursor for the morphology of the outer enamel surface (OES) through growth of the enamel cap. Hence, the unworn OES morphology results from a sequence of two developmental processes generating variation upon which natural selection can act. Mammalian dental evolution furthermore entails modifications in enamel microstructure and enamel thickness as adaptations to fracture and wear resistance [7–13]. Parenthetically, enamel thickness is reliant on life history traits such as body size, lifespan and dietary behavior. This latter includes the physical properties of food items, daily feeding times and the duration of the chewing cycle.

Dental occlusal novelties are suggested to emerge mainly from modifications of the patterning cascade method of cusp development, driven by the primary and secondary enamel knots, shaping the oral epithelium and mesenchyme interface [2, 6, 14] (see also [15–16] for alternative hypotheses on the processes controlling the folding of the inner enamel epithelium). It implies that change in the OES involves modifications that occur in the EDJ morphology and that enamel is a secondary source of morphological novelty [17]. The statement whereby OES mostly reflects EDJ morphology has been supported by numerous observations [6, 18–22] but variations in enamel thickness might impact the way OES echoes EDJ morphology. Early studies have emphasized subtle differences between EDJ and OES morphologies. For instance, since the 1950s, Butler [6] has stated that “(sic) *the crown pattern of the tooth is essentially that of the surface of the dentine (dentine-enamel junction)*, *modified by the deposition of enamel which may be uneven in thickness*”. Butler [6], and following authors (see e.g., [23–26]) have already reported lack of concordance between the EDJ surface and OES for the expression of some localized features (see also [17, 20, 22, 27]). Besides, some authors distinguish between features that are generated by localized thickening of the enamel i.e. crenation, wrinkles [6, 28, 29] from those which are reflected by the surface of the dentine i.e., folds [30] and true cusps [28]. In the same manner, Butler [6] suggested that in modern humans, thick enamel “(sic) has blanketed over minor cusps and ridges so that they appear to have become absorbed into adjacent large cusps”.

Thus, understanding how the EDJ morphology is reflected and how much of it is reflected in the OES is a major issue in assessing the relative contribution of these two organs to morphological variation and their respective response to natural selection. While previous studies emphasized on site-specific, localized differences between OES and EDJ such as crest, tubercle, crenation, wrinkles (e.g. [21, 26–27, 31–34]), we choose to first assess how and to what extent EDJ topography is reflected in the OES by quantifying the relative contribution of these two organs to primate upper molar overall morphological variation.

**Fig 1:**
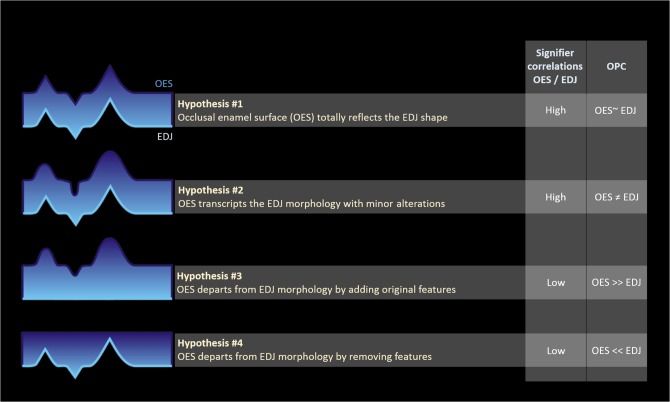
Working hypotheses on the relationships between OES/EDJ morphologies OES, occlusal enamel surface; EDJ, enamel-dentine junction; OPC, orientation patch count The four hypotheses on how the OES echoes EDJ morphology are considered Following hyp^#1^ the correlation between the EDJ and the OES is expected to be total and both organs are expected to show the same amount of occlusal complexity (OPC); hyp^#2^, the correlation between the EDJ and the OES is expected to be high and both organs are expected to show different amount of occlusal complexity; hyp^#3^, the correlation between the EDJ and the OES is expected to be low and the OES is expected to show greater amount of occlusal complexity; hyp^#4^, the correlation between the EDJ and the OES is expected to be low and the OES is expected to show lower amount of occlusal complexity.

Consideration will be given to the following hypotheses on how the OES echoes EDJ morphology: hyp^#1^, the OES completely reflects EDJ morphology; hyp^#2^, the OES transcribes EDJ morphology with minor alterations; hyp^#3^, the OES departs from EDJ morphology by adding original features such as cusps, cusplets, crests, crenulations i.e., any morphological elements affecting the complexity of the occlusal crown morphology in the sense of [17]; hyp^#4^, the OES departs from EDJ morphology by removing features. Each of these hypotheses can be tested through the analysis of correlation levels and occlusal complexity variations between the EDJ and the OES (Fig 1). For this purpose, the OES and the EDJ will be comprehensively quantified and their relationships assessed by means of 3D Dental Topometry in a set of virtual models of primate upper second unworn to slightly worn molars.

## Materials and Methods

### Material

The sample includes 29 primate taxa distributed across 76 individuals. The studied molars belong to the osteological collections of the iPHEP/University of Poitiers (France), the Department of African Zoology, the Royal Museum of Central Africa (Tervuren, Belgium) and to the National Museum of Natural History (Paris, France). The S1 Table lists all specimens, their provenance and their reference numbers. While most of the selected molars are unworn, few present light cuspal wear. However, wear facets represent less than 5% of the total area for those teeth. The sample covers a wide range of body sizes as demonstrated by tooth size and dietary habits as demonstrated by tooth shape and the level of enamel thickness, two aspects which are of major importance when considering the relationship between the OES and the EDJ.

### Three-dimensional μCT-scans and 3D surfaces

High-resolution micro-computed tomography (HR-μCT) images taken of the original molars were initially used to compute virtual 3D models from which the enamel cap can be isolated. Each molar was scanned with Viscom X1050 or EasyTom HR-microtomographs at between 10 and 30 μm isovoxel resolution, depending on the tooth size. Virtual 3D models were extracted from μCT image stacks and the enamel cap was isolated from the dentine tissue using both automated and manual processing. The crown enamel volume data was converted into a polygonal surface, a 3D irregular array of contiguous triangles corresponding to a set of tridimensional points or nodes connected by edges. This operation allowed the partition of the crown enamel into its inner (EDJ) and outer (OES) components. For analysis purposes and in order to minimize the computational load, each EDJ and OES was set to an equivalent amount of 55k polygons by a re-tessellation of the original polyhedral surface with tooth-size standardization of the polygonal unit area (i.e., each surface is constituted by polygons of equivalent area, which area depends of the tooth size) [35].

The OES and EDJ were treated as two dependent 3D polygonal surfaces. For all molars, the three-dimensional position and orientation of the OES/EDJ couple was standardized using original best-fit procedure applied to the occlusal molar basin for (xy) alignment, then alignment of the axis formed by paracone-protocone dentine horn tips with the 3D-space x-axis and translation of the surfaces so that the lowest point of the virtual representation of the molar cervix is set to z = 0 (see S1A–S1G Fig). With the molars aligned, a sub-sampling was performed of OES and EDJ occlusal surfaces as the regions above a plane parallel to the (xy) reference plane and passing respectively by the lowermost point of i) the occlusal enamel basin for the OES, and ii) the enamel-dentine junction basin for the EDJ (see S1H Fig).

A set of four topometrical signifiers, namely elevation, inclination, orientation and mean curvature, was then retrieved for each of the approximately 55k polygons respectively constituting the OES and EDJ 3D surfaces using routines and program developed in-lab [35]. Orientation is used to compute the 3D Orientation Patch Count (OPC) as the number of patches each constituted by a set of contiguous triangles sharing a similar orientation range [36]. The orientation intervals are set to 45° producing eight orientation groups. Furthermore, the 3D-enamel thickness (3DET) was computed for each molar as the minimum normal Euclidean distance from each OES polygon to the first EDJ polygon intersected [35]. The resulting list of OES/EDJ coupled-polygons was stored and used as a connectivity matrix to correlate OES and EDJ signifiers [35]. In the present analysis, the dataset is restricted to the occlusal sub-volumes of the OES and the EDJ, directly involved in food processing and tooth occlusion (see S1H Fig).

### Data analysis

For each molar, the connectivity matrix was used to compute the OES/EDJ coupled-polygons topometrical signifier matrix corresponding to about 25k values per signifier for the OES, which translates to one *elevation*, *orientation*, *inclination*, *mean curvature* and area value for each OES occlusal polygon, and about 25k values for each corresponding EDJ polygon signifier. Also, the 3D-enamel thickness (3DET) value is attached to each OES/EDJ coupled-polygon yielding a matrix totalizing about 250k values per molar. For each *elevation*, *orientation*, *inclination* and *mean curvature* coupled-dataset, 100 random samplings of 250 coupled-values each were drawn. The correlation coefficient (r) and associated significance p value have been computed for each of these bootstrapped samples allowing the computation of average and minimum/maximum correlation levels for all comparisons within each topometrical signifier for each molar. The interval of correlation levels better takes into account the respective contribution of topographically complex regions and featureless regions in the computation of the average coefficient of correlation.

The 3D enamel thickness dataset computed for each molar has been normalized using a size-standardization procedure. Each couple of OES/EDJ surfaces has been resized with the isometric scale so that all the specimens present a similar OES 3D area. The computed scale factor was then applied to each 3DET dataset in order to obtain standardized 3D enamel thickness (3DET^STD^).

## Results

**Fig 2:**
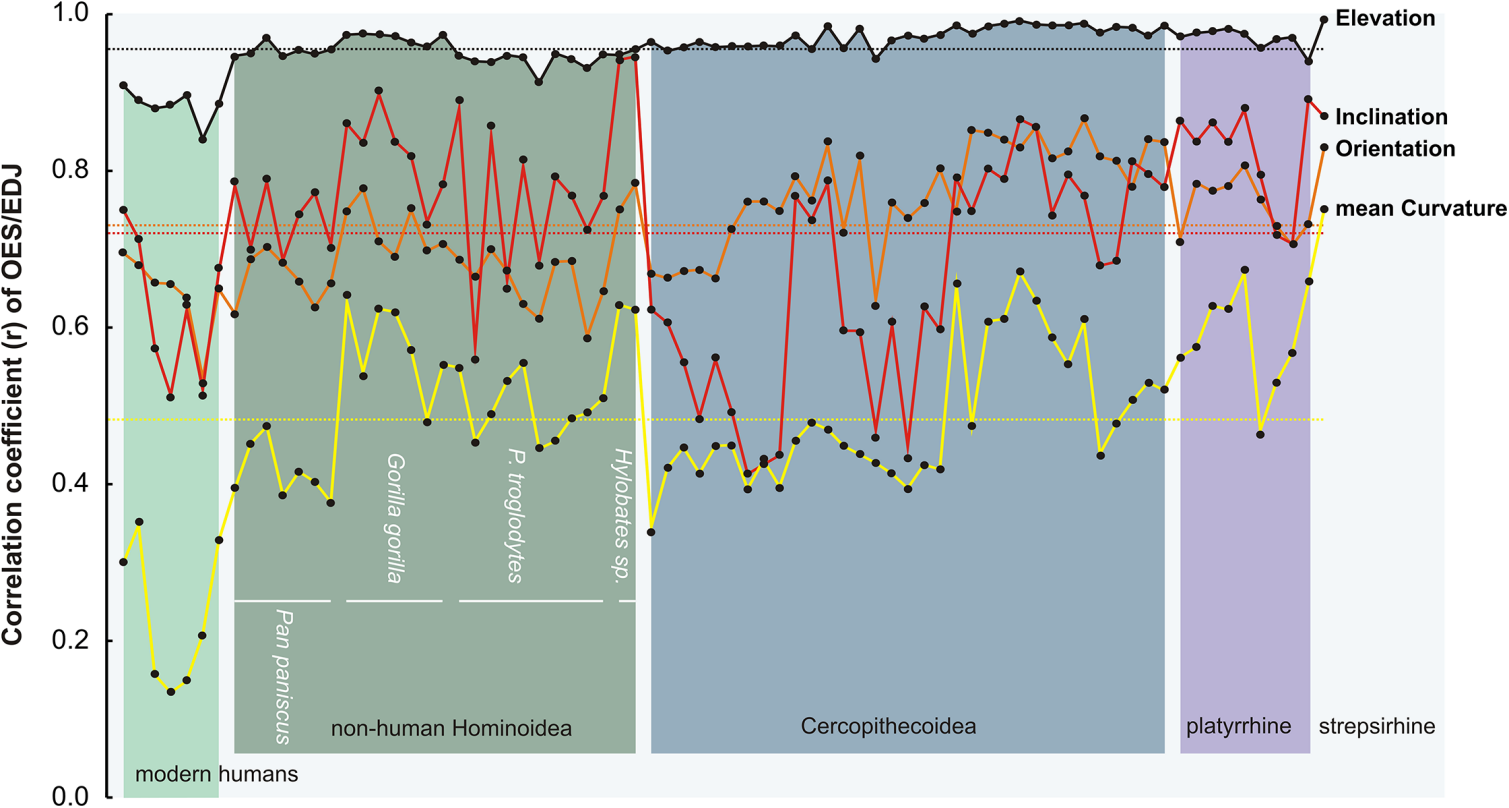
Level of correlation between EDJ and OES for topometrical signifiers within the primate sample The correlation coefficient (r) is displayed for elevation (black profile), inclination (red), orientation (orange) and mean curvature (yellow) for each specimen (black dot) Dotted lines correspond to the average correlation level for each topometrical signifier.

Fig 2 presents the correlation profiles for the four topometrical variables within the primate sample. There was a significant correlation of elevation, orientation and inclination between the OES and the EDJ for all taxa, with the highest correlation values being recorded for elevation (Fig 2 and see S2 Table). The level of correlation was lower for orientation and inclination, both parameters showing comparable r values. The lowest level of correlation was recorded for mean curvature, with no significance in four modern human individuals, from 7% to 47% of the total number of computed correlations depending on specimens, (see S2 Table).

The very high correlation of elevation revealed that, overall, EDJ deeply constraints OES topography. While a small amount of variation may still be produced by enamel, this strong correlation level in our sample contradicts hyp^#3^ and hyp^#4^. Levels of correlation for both orientation and inclination were high. On average, orientation presented slightly lower correlation values but this topometrical signifier is angular and measured on 360° implying that very close points may be coded on x and x+360°. Both orientation and inclination indicated the presence of an EDJ nucleus for each OES feature, such as cusps, crests and ridges, but above all highlighted the fact that enamel modifies, in some way, the EDJ topography for these signifiers. Such results in primates contradict hypotheses hyp^#1^, hyp^#3^ and hyp^#4^, and support hypothesis hyp^#2^. Considering mean curvature, which is a measure of inclination variation (see angularity in Ungar and Williamson [37]), weak to moderate correlations were recorded within the sample. This implies that, for this signifier, enamel may notably modify the OES topography in relation to EDJ albeit not in the same manner from one taxon to another. This contradicts hypothesis hyp^#1^. Mean curvature varies from negative values in strictly concave regions to positive values in strictly convex regions. Also, the levels of mean curvature values provide a quantitative assessment of the pattern of convexity/concavity. Slightly concave or smooth convex regions, for example with a rounded cusp, have low mean curvature values whereas highly concave or sharp convex regions such as grooves or crests show mean curvature values with the highest levels. Regions where a positive and negative principal curvature cancel each other out, namely flat regions, present mean curvature values equal or close to zero.

The computed mean curvature values tend to show a ‘blunting effect’ from enamel on the EDJ [21]. The highly convex EDJ values were coupled with moderately convex OES values, which induced a low correlation. In modern humans with thick enamel this pattern was supplemented by a grooving effect. The moderately concave EDJ values were coupled with highly concave values on the OES (see S2 Fig). Correlation results demonstrated weak to moderate variation from the EDJ to the OES, refuting hypothesis hyp^#^1 that there was no full, albeit significant, transcription of EDJ morphology onto the OES in terms of orientation, inclination or mean curvature. A partial correlation questioned the potential of an enamel cap to increase or decrease the EDJ variation on the OES and its potential to generate or buffer dental novelty on the OES in an evolutionary context.

In order to assess whether enamel adds features (crenations) to the OES in relation to the EDJ, we computed the OPC for each individual EDJ and OES [36]. The OPC is a measure of occlusal complexity (a measure reflecting the amount of morphological features) which, it is suggested, reflects dietary specializations [17, 36, 38–40]. In this primate sample, the OES appears to have a high correlation to the EDJ (r = 0.819, p<0.001; see S3 Fig and S3 Table). The occlusal enamel surface presents an OPC that is significantly higher than the EDJ (Wilcoxon test Z = 4.61, p<0.001) with the exception of some modern humans, gibbons and one common marmoset. The level of correlation recorded in this analysis largely exceeded that computed using the Skinner et al. [17] dataset. The correlation is r = 0.70 using first and second lower molars of thirteen extinct and extant anthropoid taxa, mostly hominoids, in Skinner et al. [17]. This correlation level falls to r = 0.44 when the *Pongo* outlier is removed from the sample. Such results may reflect methodological differences with a 2.5D versus 3D OPC, but it is also due to sampling since only the OES and the EDJ from the same individual are compared in this study while the Skinner et al. [17] sample includes multi-individual comparisons. Besides, one can consider that results may differ according to the tooth position under consideration. Hence, for instance, Smith et al. [41] demonstrated a trend of increasing enamel thickness from anterior to posterior teeth in modern humans and chimpanzees. Such a trend might also apply to the OPC OES/EDJ relationships, and require further investigation. Results suggest that the EDJ constrains OES occlusal complexity but slight enamel variations may produce higher OPC diversity on the OES.

Skinner et al. [17] identified three different patterns of OPC relationships between OES and EDJ within primates. First they identify one pattern in which OPC^OES^ equals or slightly differs from OPC^EDJ^ (e.g. hominins). This pattern corresponds to our hyp #^1–2^. They distinguish a second pattern in which OPC^OES^ is higher than OPC^EDJ^ (e.g. *Pan*, *Pongo*), without adding original morphological features. This second pattern corresponds to our hyp #^2^. Skinner et al. [17] suggest that in this case “(sic) the increase in morphological features via enamel deposition is best characterized as an expansion of features already present at the EDJ”. The third pattern is solely represented by genus *Chiropotes (Pitheciidae)* which exhibits much higher OPC^OES^ than OPC^EDJ^ in association with original enamel features not reflected on the EDJ (wrinkles). This third pattern corresponds to our hyp #^3^.

In our study, the pattern 1 of Skinner et al. [17] is predominantly represented (i.e., OPC^OES^/OPC^EDJ^ around 1 and inferior to 1.50). In this pattern, only the thick-enameled modern humans (7 individuals) and the genus *Hylobates* (2 individuals) tend to exhibit slightly higher OPC values at EDJ than at OES (modern human ratio OPC^OES^/OPC^EDJ^ = 0.90). The pattern 2 (i.e., OPC^OES^/OPC^EDJ^ around 1.50 and inferior to 2.30) is found for a few *Pan troglodytes* individuals, and some representatives of *Lemur*, cercopithecid, and *Lagothrix* taxa (S4 Fig). No taxa in our sample exhibits the pattern observed in *Chiropotes*, including our sampled Pitheciidae *Callicebus cupreus*.

Overall, our results on OPC contradict hypothesis hyp^#1^ and hyp^#4^ considering the significantly higher OPC value on OES than on EDJ, and should contradict hyp^#3^ and hyp^#4^ considering the high level of recorded correlation between OES and EDJ (Fig 1).

The data supports hypothesis hyp^#2^ even though mean curvature and the OPC aspects of the tooth shape did not make it possible to formally discard hypothesis hyp^#3^ especially considering the absence of *Chiropotes* in our study. One corollary question would be how the level of enamel thickness impacts the way the OES mirrors the EDJ topometrical signifiers, especially the mean curvature and the OPC.

**Fig 3:**
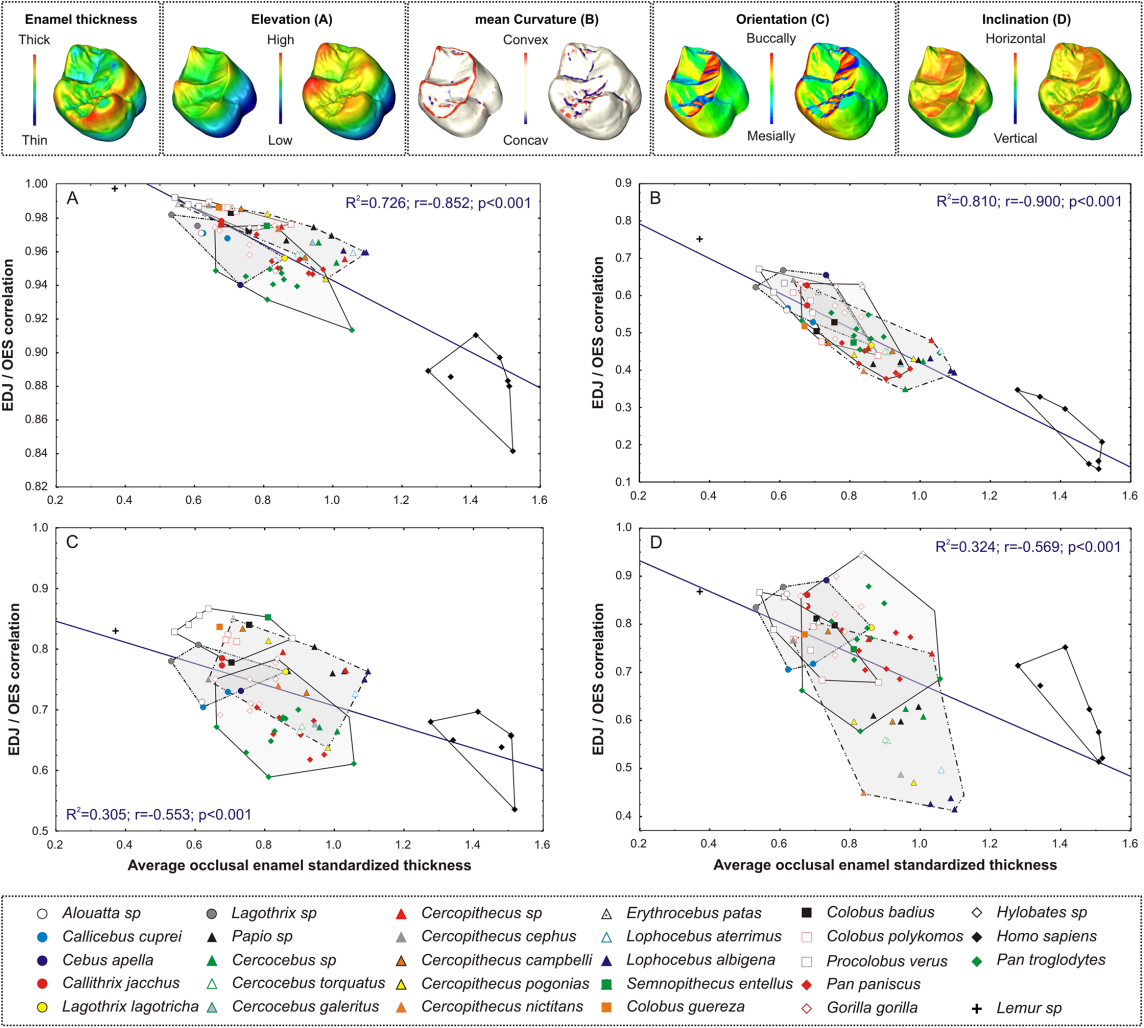
Topometrical individual OES/EDJ correlations relative to average occlusal enamel thickness (mm) of size standardized molars (A) Elevation (B) Mean Curvature (C) Orientation (D) Inclination The tridimensional maps (upper row) illustrate OES (right) and EDJ (left) topometrical data and enamel thickness data (left corner box) used for computing correlations (gorilla molar is shown). For visualization purpose, only extreme concave and convex values are shown on Mean Curvature tridimensional maps. Convex hulls delimit respectively platyrrhines (circles), cercopithecoids (triangles), non-human hominoids (diamonds) and modern humans (black diamonds) The coefficients of determination (R2), correlation (r) and p-value are given for each regression line between the EDJ/OES correlation and 3DET^STD^ (blue) 3DET^STD^, average occlusal enamel thickness of size standardized molars.

Intra-individual OES / EDJ correlations of topometrical signifiers have been regressed on average occlusal enamel thickness (3DET) of size standardized molars (3DET^STD^) for each topometrical signifier (Fig 3). There is a significant moderate to high anti-correlation for all comparisons, with the highest correlation being for mean curvature and elevation. Although relationships between the EDJ and OES elevations decrease in taxa with thick enamel, the correlation remains strong (r>0.8). Conversely, enamel thickness significantly alters the relationships between EDJ and OES curvature with a non-significant correlation in some modern humans with thick enamel. The variation of enamel thickness has a low impact on the OES/EDJ correlation for orientation and inclination. Hence, enamel thickening preserves the EDJ occlusal pattern but also the EDJ occlusal shape, except for the mean curvature. However, the blunting of the EDJ is nothing more than a mere geometrical consequence of the homogeneous growth of the enamel cap, necessarily enlarging the occlusal surface and the tip of the dentine horns (see e.g., S5 Fig). This is also responsible for the lower correlation between the OES and the EDJ in taxa with thicker enamel. Such pattern could also be linked to enamel prism diameter enlargement near the cusp tips [42, 43] and/or directly involve EDJ occlusal morphology including dentine horns relative height and dentine horns spacing (see e.g. [44]).

Considering occlusal complexity, the ratio of OES and EDJ OPCs has been regressed on 3DET^STD^ (see S4 Fig) and shows a non-significant correlation (r = -0.188, p>0.05).

## Discussion

These results support hypothesis hyp^#2^ that the OES transcribes the EDJ morphology with minor or localized geometrical alterations without adding or removing morphological features to the EDJ at least for the taxa under consideration. This outcome could be of major importance in interpreting dental evolution in primate lineages. It may, for instance, challenge the relevance of considering independent discrete enamel and dentine characters in cladistic analyses (e.g. in hominins, see [19–21, 31]). Besides, understanding the way natural selection acts on the enamel cap and the EDJ in the context of the emergence and fixation of dental novelties is a major issue in paleoprimatology. It is an issue which is now within easy reach. The OES morphologies and enamel thickness variations of the teeth are commonly viewed as adaptations to particular diets. On the one hand, OES occlusal morphology consists of tools including pestle, mortar, blade and shearing crest, used to process food items which are associated with slicing, crushing, grinding or puncturing functions (e.g. [45–47]). On the other hand, the enamel cap ensures mainly protective functions such as resistance to tooth wear and fracture [48–49]. Consequently, enamel thickness is reliant on life history traits such as body mass since larger primates tend to have thicker enamel [50] (see also [51] for the relationship between body size and tooth sharpness in mammals), lifespan and lifetime dietary wear [49], dietary behavior and by extension ecological niches (e.g. [52]). In this context the protective function of the enamel cap is reliant on the physical properties of food items, daily feeding times, lifespan and the nature and duration of the chewing cycle. Moreover, variation of enamel thickness distribution over the tooth crown relies, in part, on the differential functional demands on enamel cap regions during mastication [53]. From an evolutionary perspective it should be considered that selective pressures might apply on dental tools and occlusal complexity and/or enamel protective functions. Such a notion should be central in reassessing morphological diversity in the primate fossil record and serve to interpret observed discrepancies between inherited dental morphologies and effective diets. (e.g. [54–57]). However, Suwa and Kono [43] proposed additional hypothesis to explain enamel thickening variation over the tooth crown. These authors invokes a developmental mechanism i.e., a distribution of cell–cell tension and/or compression within the outward-migrating ameloblast sheet, which would limit enamel thickness in specific areas, most likely associated with steepness variation of EDJ topography at and around the cusp tips. While investigating such hypothesis is beyond the scope of the present article, preliminary analyses of the relationship between EDJ inclination and the enamel thickness do not reveal significant correlation in our sample.

The results show that enamel thickening does not directly influence higher OES complexity. This proves the dissociation between the morphological adaptive response to consumed food and the mechanical response to the physical properties of food items in terms of resistance to tooth wear and fracture. This study on the influence of enamel thickness on OES/EDJ relationships implies that the main possible counter-selection to enamel thickening is a specific food-processing requirement, be it puncturing or slicing, which requires sharp or pointed dental tools. In the course of evolution, there is a balance between the necessity to preserve such dental tools and the necessity to protect the tooth from wear and fracture by thickening the enamel cap. This is emphasized by the fact that all the extant primates displaying pointed or sharp tooth occlusal surfaces have thin enamel. Hence, the morphology of the primate OES could result from a selection tending towards improved tooth resistance. This in turn implies a higher level of enamel thickness [48, 58] compatible with the preservation of a relative efficiency of the dental tools. This morphology could also result from a selection tending towards efficient dental tools, through either counter-selection of a thick enamel cap or the selection of enamel thickness levels entailing new functionalities for dental features.

Most of the tooth shape is supported by the EDJ. This tissue ensures functional occlusion by determining the occlusal pattern and constrains the principal OES topographical properties such as elevation, inclination and orientation. Therefore, the EDJ is the main source of novelty in the occlusal pattern in primates, i.e., origination of new dental traits such as cusplets, crests, and cingula. The enamel cap does not seem to generate novelties in this manner in our sample, i.e. ‘crenations’ in the sense of Butler [6]. However, such crenations have been reported by previous studies (e.g. [17, 20, 25–27, 31]), which implies the possibility that enamel cap can be a secondary source of novelty cannot be excluded. As is, enamel thickness variations modify the OES topography and may potentially modify dental functionalities, i.e. the functional state of EDJ dental traits. Our result confirm Skinner et al. [17]’s statement according to which “(sic) enamel deposition modifies traits, rather than eliminating or generating them”. A dentine horn may give rise either to a pointed or a bulbous cusp depending on both the relative elevation of the dentine horn (see also [44]) and enamel thickness. A dentine valley may give either to a valley or a groove/fissure [6] on OES depending on the dentine relief spacing and enamel thickness. In terms of natural selection, there is a balance between increasing tooth resistance to wear and fracture and maintaining efficient dental tools. In this sense the enamel cap acts as a functional buffer of the molar occlusal pattern in the course of primate evolution.

## Summary Conclusion

The approach proposed in the present paper is likely to improve our understanding of the respective influences of the enamel-dentine junction and the enamel layer over crown morphology. The selected topometrical signifiers are shown here to be useful for characterizing tooth form and dental tissue relationships. Also, though several studies have recently undertaken three-dimensional analyses of the tooth structural morphology (e.g. [13, 19–21]), our results introduced for the first time, to our knowledge, the correlations between enamel occlusal surface and enamel-dentine junction using tridimensional topometrical parameters and their geometric relationship.

Our results confirm that, for the primate second upper molar, the enamel-dentine junction carries most of the occlusal morphology of the tooth, as there is a significant correlation for elevation, orientation, inclination, curvature and occlusal complexity between the outer enamel surface and the enamel dentine junction for all studied primate taxa with the exception of four modern humans for curvature. Moreover, we show that, the enamel layer thickness influences the relationship between OES and EDJ for all selected topometrical parameters apart from occlusal patch count, the recorded correlations significantly decreasing along with enamel thickening in our sample.

## Supporting Information

S1 Fig Molar orientation protocol. Example of a *Gorilla* second upper molar (A) Isolated occlusal enamel (OES, left panel) and enamel-dentine junction (EDJ, right panel) un-oriented surfaces in 3D space (B) The EDJ occlusal basin surface (orange) is isolated from the crown (C) A plane (p) is produced from the EDJ basin using Best Fit (least square) procedure (D) The plane (p) is attached to the original complete EDJ surface (D1), an axis (Pa-Pr) passing through the dentine horn tips corresponding to the paracone and protocone is created (the axis points towards protocone (D2) (E) The OES and EDJ surfaces are re-aligned to 3D space with plane (p) parallel to (xy) and (Pa-Pr) axis parallel to x and pointing toward x positive The lowermost point of the occlusal basin is set to z = 0 (F) Distal view of the aligned molar (OES and EDJ) (G) Lingual view of the aligned molar (OES and EDJ) (H) The OES and EDJ occlusal surfaces correspond to the regions above a plane parallel to the (xy) reference plane and passing respectively by the lowermost point of the occlusal enamel basin for the OES, and the enamel-dentine junction basin for the EDJ, colored in red.
(TIF)

S2 Fig Schematic illustration of curvature modification from enamel-dentine junction (EDJ) to occlusal enamel surface (OES). Examples of upper second molar of *Cercocebus torquatus* (A), *Homo sapiens* (B) and *Callicebus cupreus* (C) Higher curvature levels (convexity) occur at the EDJ (upper row), while curvature is lowered at the OES (lower row) On the contrary, greater concavity (negative curvature) occurs at the OES while concavity is slight at the EDJ. For visualization purpose, color maps only show extreme convexity (red) and extreme concavity (blue) values.
(TIF)

S3 Fig Orientation patch count (OPC), relationship between enamel-dentine junction (EDJ) and occlusal enamel surface (OES). Convex hulls delimit respectively platyrrhines (circles), cercopithecoids (triangles), non-human hominoids (diamonds) and modern humans (black diamonds) The coefficient of determination (R^2^), correlation (r) and p-value is given for the regression line between OPC^EDJ^ correlation and OPC^OES^ (blue line). See Fig 3 caption for legend.
(TIF)

S4 Fig Orientation patch count ratio of OES/EDJ and its relationship to average occlusal enamel standardized thickness. Convex hulls delimit respectively platyrrhines (circles), cercopithecoids (triangles), non-human hominoids (diamonds) and modern humans (black diamonds) The coefficient of determination (R^2^), correlation (r) and p-value is given for the regression line between OPC^OES^/OPC^EDJ^ and 3DET^STD^ (blue line) 3DET^STD^, average occlusal enamel thickness of size standardized molars; OPC, orientation patch count. See Fig 3 caption for legend.
(TIF)

S5 Fig Model simulation showing the geometric effect that produces concavity while enamel thickness increases. Schemes from A1 to G1 (posterior view) and A2 to G2 (superior view) illustrate the increasing level of deformation of a plane which models increasing enamel thickness. At higher enamel thickness, the geometry imposes the formation of concavities (F2-G2; blue).
(TIF)

S1 Table Molar Sample. Our method is applied to a set of 76 unworn to slightly worn upper second molars, the specimens are housed in European institutions listed as iPHEP, Institut de Paléoprimatologie et Paléontologie Humaine, Evolution et Paléoenvironnements (Université de Poitiers—Faculté des Sciences, France); MNHN, Musée National d’Histoire Naturelle (Paris, France); RMCA, Musée Royal d’Afrique Centrale (Tervuren, Belgium).
(DOCX)

S2 Table Correlation (bootstrapped r coefficients) between OES and EDJ occlusal signifiers. Min, minimum; Max, maximum; NS non-significant Molar specimens belong to *H*. *sapiens* (Hsap), *Pan paniscus* (Ppan), *Gorilla gorilla* (Ggor), *Pan troglodytes* (Ptro), *Hylobates sp*. (Hyl), *Cercocebus sp*. (Cerbsp), *Cercocebus torquatus* (Cerbtor), *Cercocebus galeritus* (Cerbgal), *Lophocebus aterrimus* (Lophat), *Lophocebus albigena* (Lophalb), *Cercopithecus sp*. (Cercsp), *Cercopithecus campbelli* (Cerccamp), *Cercopithecus pogonias* (Cercpog), *Cercopithecus cephus* (Cerccep), *Cercopithecus nictitans* (Cercnic), *Erythrocebus patas* (Erythpat), *Papio sp* (Papsp), *Semnopithecus entellus* (Semenent), *Procolobus verus* (Procver), *Colobus polykomos* (Colpol), *Colobus badius* (Colbad), *Colobus guereza* (Colguer), *Alouatta sp*. (Allousp), *Callithrix jacchus* (Calljac), *Lagothrix sp*. (Lagsp), *Lagothrix lagotricha* (Laglag), *Callicebus cupreus* (Callcup), *Cebus apella* (Cebap), *Lemur sp*. (Lemsp).
(DOCX)

S3 Table Computed occlusal patch count (OES and EDJ) and enamel thickness variables for the primate molar sample. 3DET, average 3D occlusal thickness (mm); 3DET^STD^, average standardized 3D occlusal thickness; 3DOPC^OES^, 3D orientation patch count for the OES; 3DOPC^EDJ^, 3D orientation patch count for the EDJ. See S2 Table for specimen names.
(DOCX)
