# Supplementary material for: To What Extent is Primate Second Molar Enamel Occlusal Morphology Shaped by the Enamel-Dentine Junction?
Source: PLoS One. 2015 Sep 25;10(9):e0138802. doi: 10.1371/journal.pone.0138802 (PMC4634312; doi:10.1371/journal.pone.0138802)
Supplement: S1 Table — Our method is applied to a set of 76 unworn to slightly worn upper second molars, the specimens are housed in European institutions listed as iPHEP, Institut de Paléoprimatologie et Paléontologie Humaine, Evolution et Paléoenvironnements (Université de Poitiers—Faculté des Sciences, France); MNHN, Musée National d’Histoire Naturelle (Paris, France); RMCA, Musée Royal d’Afrique Centrale (Tervuren, Belgium). (DOCX) [file pone.0138802.s006.docx]

**S1 Table. Molar Sample.** Our method is applied to a set of 76 unworn to slightly worn upper second molars, the specimens are housed in European institutions listed as iPHEP, Institut de Paléoprimatologie et Paléontologie Humaine, Evolution et Paléoenvironnements (Université de Poitiers - Faculté des Sciences, France) ; MNHN, Musée National d’Histoire Naturelle (Paris, France); RMCA, Musée Royal d’Afrique Centrale (Tervuren, Belgium).

**S1 Table**

| **Family/Subfamily** | **Taxon** | **Count** | **Source** | **Specimen number** |
| --- | --- | --- | --- | --- |
| Lemuridae | *Lemur sp.* | **1** | iPHEP | #M19-Lem-3 |
| Atelidae | *Alouatta sp.* | **1** | iPHEP | #M19-Alo-2 |
| Atelidae | *Lagothrix sp.* | **2** | iPHEP | #M19-Lg-A ; -Lg-B |
| Atelidae | *Lagothrix lagotricha* | **1** | MNHN | #1934-1019 |
| Callitrichinae | *Callithrix jacchus* | **2** | MNHN | #64-232a ; #64-232b |
| Cebinae | *Cebus apella* | **1** | MNHN | #305 |
| Pithecidae | *Callicebus cupreus* | **2** | MNHN | #20-92a ; #20-92b |
| Cercopithecinae | *Cercocebus sp.* | **2** | iPHEP | #M19-Cb-2 ; -Cb-3 |
| Cercopithecinae | *Cercocebus galeritus* | **1** | RMCA | #14486 |
| Cercopithecinae | *Cercocebus torquatus* | **2** | RMCA | #8107M44a ; -b ; |
| Cercopithecinae | *Cercopithecus campbelli* | **2** | RMCA | #80028M24 ; #36280 |
| Cercopithecinae | *Cercopithecus pogonias* | **2** | RMCA | #18273 ; #15595 |
| Cercopithecinae | *Cercopithecus cephus* | **1** | RMCA | #17507 |
| Cercopithecinae | *Cercopithecus nictitans* | **1** | RMCA | #15650 |
| Cercopithecinae | *Erythrocebus patas* | **1** | RMCA | #8629 |
| Cercopithecinae | *Cercopithecus sp* | **2** | iPHEP | #M19-Cc-1 ; -Cc-3 |
| Cercopithecinae | *Lophocebus albigena* | **3** | RMCA | #83006M276 ; #90042M301a ; -M301b |
| Cercopithecinae | *Lophocebus aterrimus* | **1** | RMCA | #14113 |
| Colobinae | *Procolobus verus* | **3** | RMCA | #86002M50 ; -M34 ; -M48 |
| Colobinae | *Colobus polykomos* | **5** | RMCA | #38158 ; #07M174 ; #10602, -308, -548 |
| Colobinae | *Colobus badius* | **2** | RMCA | #91060M57 ; #83042M77 |
| Colobinae | *Colobus guereza* | **1** | RMCA | #1216 |
| Colobinae | *Semnopithecus entellus* | **1** | MNHN | #950-90-55 |
| Papioninae | *Papio sp* | **3** | RMCA/iPHEP | M19-3 ; M19-4 ; RMC1 |
| Hominidae | *Gorilla gorilla* | **3** | RMCA | #15355 ; #27755 ; #75056M15 |
|  |  | **4** | iPHEP | #PM321 ; #8311 ; #PM319 ; #M19G-1 |
| Hominidae | *Pan paniscus* | **7** | RMCA | #29065b, #28712, #29029 ; #29032 ; |
|  |  |  |  | #29041a; #29053 ; #29062a |
| Hominidae | *Pan troglodytes* | **7** | RMCA | #1942 ; #2917 ; #23508 ; #23511b ; |
|  |  |  |  | #73018M5 ; #26967a ; #6043 |
|  |  | **3** | iPHEP | #M19-5-50d ; #M19-5-51 ; #M19-5-52 |
| Hominidae | *Homo sapiens* | **7** | iPHEP | #185-141 ; #M19-H1 ; -H2 ; -H3 ; -H4 ; |
|  |  |  |  | -H5 ; -H6 |
| Hylobatidae | *Hylobates sp.* | **2** | iPHEP | #M19-Hy-1a ; -1b |
|  | **Total** | **76** |  |  |
