# Supplementary material for: To What Extent is Primate Second Molar Enamel Occlusal Morphology Shaped by the Enamel-Dentine Junction?
Source: PLoS One. 2015 Sep 25;10(9):e0138802. doi: 10.1371/journal.pone.0138802 (PMC4634312; doi:10.1371/journal.pone.0138802)
Supplement: S2 Table — Min, minimum; Max, maximum; NS non-significant Molar specimens belong to H. sapiens (Hsap), Pan paniscus (Ppan), Gorilla gorilla (Ggor), Pan troglodytes (Ptro), Hylobates sp. (Hyl), Cercocebus sp. (Cerbsp), Cercocebus torquatus (Cerbtor), Cercocebus galeritus (Cerbgal), Lophocebus aterrimus (Lophat), Lophocebus albigena (Lophalb), Cercopithecus sp. (Cercsp), Cercopithecus campbelli (Cerccamp), Cercopithecus pogonias (Cercpog), Cercopithecus cephus (Cerccep), Cercopithecus nictitans (Cercnic), Erythrocebus patas (Erythpat), Papio sp (Papsp), Semnopithecus entellus (Semenent), Procolobus verus (Procver), Colobus polykomos (Colpol), Colobus badius (Colbad), Colobus guereza (Colguer), Alouatta sp. (Allousp), Callithrix jacchus (Calljac), Lagothrix sp. (Lagsp), Lagothrix lagotricha (Laglag), Callicebus cupreus (Callcup), Cebus apella (Cebap), Lemur sp. (Lemsp). (DOCX) [file pone.0138802.s007.docx]

**S2 Table. Correlation (bootstrapped r coefficients) between OES and EDJ occlusal signifiers.** Min, minimum; Max, maximum; NS non-significant Molar specimens belong to *H. sapiens* (Hsap), *Pan paniscus* (Ppan), *Gorilla gorilla* (Ggor), *Pan troglodytes* (Ptro), *Hylobates sp.* (Hyl), *Cercocebus sp.* (Cerbsp), *Cercocebus torquatus* (Cerbtor), *Cercocebus galeritus* (Cerbgal), *Lophocebus aterrimus* (Lophat), *Lophocebus albigena* (Lophalb), *Cercopithecus sp.* (Cercsp), *Cercopithecus campbelli* (Cerccamp), *Cercopithecus pogonias* (Cercpog), *Cercopithecus cephus* (Cerccep), *Cercopithecus nictitans* (Cercnic), *Erythrocebus patas* (Erythpat), *Papio sp* (Papsp), *Semnopithecus entellus* (Semenent), *Procolobus verus* (Procver), *Colobus polykomos* (Colpol), *Colobus badius* (Colbad), *Colobus guereza* (Colguer), *Alouatta sp.* (Allousp), *Callithrix jacchus* (Calljac), *Lagothrix sp.* (Lagsp), *Lagothrix lagotricha* (Laglag), *Callicebus cupreus* (Callcup), *Cebus apella* (Cebap), *Lemur sp.* (Lemsp).

**S2 Table (part 1/2)**

|  | **Elevation** | | | **Orientation** | | | **Inclination** | | | **Mean curvature** | | | |
| --- | --- | --- | --- | --- | --- | --- | --- | --- | --- | --- | --- | --- | --- |
|  | **Min.** | **Mean** | **Max.** | **Min.** | **Mean** | **Max.** | **Min.** | **Mean** | **Max.** | **Min.** | **Mean** | **Max.** | **NS** |
| **Hsap_#1** | 0.89 | 0.91 | 0.93 | 0.53 | 0.70 | 0.84 | 0.68 | 0.75 | 0.82 | 0.18 | 0.30 | 0.41 | - |
| **Hsap_#2** | 0.85 | 0.89 | 0.91 | 0.50 | 0.68 | 0.87 | 0.63 | 0.71 | 0.80 | 0.24 | 0.35 | 0.47 | - |
| **Hsap_#3** | 0.83 | 0.88 | 0.91 | 0.53 | 0.66 | 0.81 | 0.49 | 0.58 | 0.66 | 0.03 | 0.16 | 0.28 | 26/100 |
| **Hsap_#4** | 0.86 | 0.88 | 0.91 | 0.49 | 0.66 | 0.81 | 0.42 | 0.51 | 0.61 | 0.00 | 0.13 | 0.27 | 47/100 |
| **Hsap_#5** | 0.87 | 0.90 | 0.92 | 0.47 | 0.64 | 0.81 | 0.51 | 0.62 | 0.69 | 0.03 | 0.15 | 0.28 | 35/100 |
| **Hsap_#6** | 0.80 | 0.84 | 0.87 | 0.37 | 0.54 | 0.80 | 0.40 | 0.52 | 0.63 | 0.07 | 0.21 | 0.33 | 7/100 |
| **Hsap_#7** | 0.84 | 0.89 | 0.91 | 0.49 | 0.65 | 0.81 | 0.60 | 0.67 | 0.75 | 0.20 | 0.33 | 0.43 | - |
| **Ppan_#1** | 0.93 | 0.95 | 0.96 | 0.49 | 0.62 | 0.78 | 0.69 | 0.78 | 0.83 | 0.22 | 0.39 | 0.54 | - |
| **Ppan_#2** | 0.94 | 0.95 | 0.96 | 0.54 | 0.69 | 0.85 | 0.63 | 0.70 | 0.78 | 0.33 | 0.45 | 0.59 | - |
| **Ppan_#3** | 0.96 | 0.97 | 0.98 | 0.57 | 0.70 | 0.84 | 0.70 | 0.79 | 0.85 | 0.37 | 0.47 | 0.57 | - |
| **Ppan_#4** | 0.93 | 0.95 | 0.96 | 0.52 | 0.68 | 0.85 | 0.61 | 0.69 | 0.77 | 0.28 | 0.39 | 0.54 | - |
| **Ppan_#5** | 0.93 | 0.95 | 0.97 | 0.50 | 0.66 | 0.84 | 0.63 | 0.75 | 0.82 | 0.26 | 0.42 | 0.55 | - |
| **Ppan_#6** | 0.93 | 0.95 | 0.96 | 0.43 | 0.63 | 0.79 | 0.68 | 0.77 | 0.84 | 0.28 | 0.40 | 0.54 | - |
| **Ppan_#7** | 0.94 | 0.96 | 0.96 | 0.48 | 0.66 | 0.84 | 0.62 | 0.71 | 0.75 | 0.22 | 0.38 | 0.48 | - |
| **Ggor_#1** | 0.97 | 0.97 | 0.98 | 0.61 | 0.75 | 0.89 | 0.81 | 0.86 | 0.91 | 0.55 | 0.63 | 0.70 | - |
| **Ggor_#2** | 0.97 | 0.98 | 0.98 | 0.60 | 0.78 | 0.92 | 0.78 | 0.84 | 0.90 | 0.39 | 0.54 | 0.64 | - |
| **Ggor_#3** | 0.97 | 0.97 | 0.98 | 0.48 | 0.71 | 0.84 | 0.86 | 0.90 | 0.93 | 0.54 | 0.62 | 0.71 | - |
| **Ggor_#4** | 0.96 | 0.97 | 0.98 | 0.49 | 0.69 | 0.86 | 0.77 | 0.84 | 0.89 | 0.54 | 0.62 | 0.72 | - |
| **Ggor_#5** | 0.95 | 0.96 | 0.97 | 0.59 | 0.75 | 0.85 | 0.76 | 0.82 | 0.87 | 0.47 | 0.57 | 0.67 | - |
| **Ggor_#6** | 0.94 | 0.96 | 0.97 | 0.49 | 0.70 | 0.81 | 0.65 | 0.74 | 0.80 | 0.35 | 0.48 | 0.58 | - |
| **Ggor_#7** | 0.97 | 0.97 | 0.98 | 0.57 | 0.71 | 0.85 | 0.70 | 0.78 | 0.86 | 0.43 | 0.55 | 0.69 | - |
| **Ptro_#1** | 0.95 | 0.95 | 0.96 | 0.51 | 0.69 | 0.80 | 0.84 | 0.88 | 0.92 | 0.45 | 0.55 | 0.64 | - |
| **Ptro_#2** | 0.92 | 0.94 | 0.96 | 0.51 | 0.67 | 0.86 | 0.46 | 0.58 | 0.66 | 0.36 | 0.46 | 0.54 | - |
| **Ptro_#3** | 0.92 | 0.94 | 0.96 | 0.53 | 0.70 | 0.85 | 0.77 | 0.84 | 0.88 | 0.37 | 0.49 | 0.59 | - |
| **Ptro_#4** | 0.93 | 0.95 | 0.96 | 0.47 | 0.67 | 0.83 | 0.55 | 0.66 | 0.75 | 0.39 | 0.53 | 0.61 | - |
| **Ptro_#5** | 0.93 | 0.95 | 0.96 | 0.48 | 0.63 | 0.80 | 0.73 | 0.81 | 0.86 | 0.43 | 0.56 | 0.63 | - |
| **Ptro_#6** | 0.88 | 0.91 | 0.93 | 0.40 | 0.61 | 0.74 | 0.53 | 0.69 | 0.76 | 0.34 | 0.45 | 0.53 | - |
| **Ptro_#7** | 0.94 | 0.95 | 0.96 | 0.54 | 0.68 | 0.83 | 0.70 | 0.79 | 0.86 | 0.33 | 0.46 | 0.55 | - |
| **Ptro_#8** | 0.92 | 0.94 | 0.96 | 0.55 | 0.69 | 0.85 | 0.70 | 0.77 | 0.83 | 0.36 | 0.48 | 0.58 | - |
| **Ptro_#9** | 0.92 | 0.93 | 0.95 | 0.36 | 0.59 | 0.77 | 0.66 | 0.73 | 0.81 | 0.37 | 0.49 | 0.62 | - |
| **Ptro_#10** | 0.92 | 0.95 | 0.96 | 0.50 | 0.65 | 0.82 | 0.69 | 0.77 | 0.82 | 0.42 | 0.51 | 0.63 | - |
| **Hyl_#1** | 0.94 | 0.95 | 0.96 | 0.59 | 0.75 | 0.91 | 0.93 | 0.94 | 0.96 | 0.50 | 0.63 | 0.71 | - |
| **Hyl_#2** | 0.94 | 0.96 | 0.96 | 0.60 | 0.78 | 0.90 | 0.93 | 0.95 | 0.96 | 0.53 | 0.62 | 0.72 | - |
| **Cerbsp_#1** | 0.95 | 0.97 | 0.97 | 0.55 | 0.67 | 0.82 | 0.53 | 0.62 | 0.71 | 0.19 | 0.35 | 0.49 | - |
| **Cerbsp_#2** | 0.94 | 0.95 | 0.97 | 0.47 | 0.66 | 0.79 | 0.51 | 0.61 | 0.74 | 0.31 | 0.42 | 0.59 | - |
| **Cerbtor_#1** | 0.95 | 0.96 | 0.97 | 0.53 | 0.67 | 0.82 | 0.42 | 0.56 | 0.67 | 0.34 | 0.45 | 0.58 | - |
| **Cerbtor_#2** | 0.95 | 0.96 | 0.97 | 0.51 | 0.66 | 0.81 | 0.34 | 0.56 | 0.66 | 0.31 | 0.45 | 0.57 | - |
| **Cerbgal_#1** | 0.96 | 0.97 | 0.97 | 0.55 | 0.67 | 0.81 | 0.34 | 0.49 | 0.63 | 0.28 | 0.41 | 0.55 | - |
| **Lophat_#1** | 0.95 | 0.96 | 0.97 | 0.58 | 0.73 | 0.84 | 0.32 | 0.49 | 0.64 | 0.33 | 0.45 | 0.57 | - |
| **Lophalb_#1** | 0.95 | 0.96 | 0.97 | 0.64 | 0.76 | 0.88 | 0.21 | 0.41 | 0.60 | 0.29 | 0.39 | 0.53 | - |
| **Lophalb_#2** | 0.95 | 0.96 | 0.97 | 0.60 | 0.76 | 0.87 | 0.27 | 0.43 | 0.62 | 0.35 | 0.43 | 0.52 | - |
| **Lophalb_#3** | 0.95 | 0.96 | 0.97 | 0.60 | 0.75 | 0.75 | 0.24 | 0.44 | 0.58 | 0.27 | 0.40 | 0.49 | - |
| **Cercsp_#1** | 0.97 | 0.97 | 0.98 | 0.60 | 0.79 | 0.91 | 0.69 | 0.77 | 0.84 | 0.34 | 0.46 | 0.56 | - |
| **Cercsp_#2** | 0.94 | 0.96 | 0.97 | 0.61 | 0.76 | 0.90 | 0.66 | 0.74 | 0.81 | 0.36 | 0.48 | 0.57 | - |
| **Cerccamp_#1** | 0.98 | 0.99 | 0.99 | 0.72 | 0.83 | 0.95 | 0.69 | 0.78 | 0.84 | 0.32 | 0.47 | 0.60 | - |
| **Cerccamp_#2** | 0.95 | 0.96 | 0.97 | 0.55 | 0.73 | 0.84 | 0.47 | 0.60 | 0.69 | 0.34 | 0.45 | 0.56 | - |
| **Cercpog_#1** | 0.98 | 0.98 | 0.99 | 0.67 | 0.81 | 0.94 | 0.45 | 0.60 | 0.70 | 0.30 | 0.44 | 0.56 | - |
| **Cercpog_#2** | 0.92 | 0.94 | 0.96 | 0.49 | 0.64 | 0.79 | 0.29 | 0.47 | 0.61 | 0.23 | 0.43 | 0.56 | - |
| **Cerccep#1** | 0.98 | 0.99 | 0.99 | 0.63 | 0.75 | 0.90 | 0.65 | 0.77 | 0.87 | 0.55 | 0.64 | 0.71 | - |
| **Cercnic#1** | 0.97 | 0.97 | 0.98 | 0.62 | 0.74 | 0.89 | 0.31 | 0.45 | 0.58 | 0.25 | 0.40 | 0.50 | - |
| **Papsp_#1** | 0.96 | 0.97 | 0.98 | 0.57 | 0.76 | 0.89 | 0.46 | 0.63 | 0.75 | 0.26 | 0.43 | 0.52 | - |
| **Papsp_#2** | 0.97 | 0.97 | 0.98 | 0.69 | 0.80 | 0.91 | 0.49 | 0.60 | 0.70 | 0.27 | 0.42 | 0.52 | - |
| **Papsp_#3** | 0.96 | 0.97 | 0.98 | 0.64 | 0.76 | 0.86 | 0.46 | 0.61 | 0.70 | 0.30 | 0.41 | 0.52 | - |
| **Erythpat#1** | 0.98 | 0.99 | 0.99 | 0.72 | 0.85 | 0.97 | 0.67 | 0.80 | 0.86 | 0.48 | 0.61 | 0.70 | - |
| **Semenent_#1** | 0.97 | 0.98 | 0.98 | 0.75 | 0.85 | 0.95 | 0.68 | 0.75 | 0.81 | 0.29 | 0.48 | 0.60 | - |
| **Procver_#1** | 0.99 | 0.99 | 0.99 | 0.72 | 0.84 | 0.96 | 0.66 | 0.79 | 0.87 | 0.53 | 0.61 | 0.70 | - |
| **Procver_#2** | 0.99 | 0.99 | 0.99 | 0.70 | 0.83 | 0.94 | 0.80 | 0.87 | 0.91 | 0.60 | 0.67 | 0.75 | - |
| **Procver_#3** | 0.98 | 0.99 | 0.99 | 0.74 | 0.86 | 0.96 | 0.78 | 0.86 | 0.92 | 0.55 | 0.64 | 0.72 | - |
| **Colpol_#1** | 0.98 | 0.99 | 0.99 | 0.66 | 0.82 | 0.94 | 0.65 | 0.75 | 0.83 | 0.48 | 0.59 | 0.68 | - |
| **Colpol_#2** | 0.98 | 0.99 | 0.99 | 0.71 | 0.82 | 0.94 | 0.70 | 0.80 | 0.87 | 0.44 | 0.55 | 0.65 | - |
| **Colpol_#3** | 0.99 | 0.99 | 0.99 | 0.74 | 0.87 | 0.95 | 0.67 | 0.77 | 0.84 | 0.50 | 0.61 | 0.74 | - |
| **Colpol_#4** | 0.97 | 0.98 | 0.98 | 0.70 | 0.82 | 0.92 | 0.54 | 0.68 | 0.77 | 0.32 | 0.44 | 0.53 | - |
| **Colpol_#5** | 0.98 | 0.98 | 0.99 | 0.67 | 0.81 | 0.91 | 0.52 | 0.68 | 0.78 | 0.33 | 0.48 | 0.58 | - |
| **Colbad_#1** | 0.98 | 0.98 | 0.99 | 0.66 | 0.78 | 0.94 | 0.75 | 0.81 | 0.87 | 0.31 | 0.51 | 0.64 | - |
| **Colbad_#2** | 0.96 | 0.97 | 0.98 | 0.68 | 0.84 | 0.93 | 0.67 | 0.80 | 0.87 | 0.41 | 0.53 | 0.63 | - |
| **Colguer_#1** | 0.98 | 0.99 | 0.99 | 0.68 | 0.84 | 0.95 | 0.70 | 0.78 | 0.87 | 0.40 | 0.52 | 0.63 | - |

| **Allousp_#1** | 0.95 | 0.97 | 0.97 | 0.56 | 0.71 | 0.86 | 0.80 | 0.86 | 0.91 | 0.43 | 0.56 | 0.70 | - |
| --- | --- | --- | --- | --- | --- | --- | --- | --- | --- | --- | --- | --- | --- |
| **Calljac_#1** | 0.97 | 0.98 | 0.98 | 0.67 | 0.79 | 0.94 | 0.76 | 0.84 | 0.90 | 0.46 | 0.58 | 0.70 | - |
| **Calljac_#2** | 0.97 | 0.98 | 0.98 | 0.59 | 0.77 | 0.89 | 0.86 | 0.86 | 0.92 | 0.51 | 0.63 | 0.76 | - |
| **Lagsp_#1** | 0.98 | 0.98 | 0.99 | 0.58 | 0.78 | 0.90 | 0.77 | 0.84 | 0.88 | 0.40 | 0.62 | 0.74 | - |
| **Lagsp_#2** | 0.97 | 0.98 | 0.98 | 0.65 | 0.81 | 0.93 | 0.81 | 0.88 | 0.92 | 0.60 | 0.67 | 0.74 | - |
| **Laglag_#1** | 0.94 | 0.96 | 0.97 | 0.63 | 0.76 | 0.93 | 0.70 | 0.79 | 0.87 | 0.37 | 0.47 | 0.62 | - |
| **Callcup_#1** | 0.96 | 0.97 | 0.98 | 0.52 | 0.73 | 0.86 | 0.60 | 0.72 | 0.79 | 0.41 | 0.53 | 0.64 | - |
| **Callcup_#2** | 0.96 | 0.97 | 0.98 | 0.61 | 0.71 | 0.82 | 0.61 | 0.71 | 0.78 | 0.47 | 0.57 | 0.68 | - |
| **Cebap_#1** | 0.92 | 0.94 | 0.95 | 0.59 | 0.73 | 0.85 | 0.86 | 0.89 | 0.93 | 0.56 | 0.66 | 0.74 | - |
| **Lemsp_#1** | 0.99 | 1.00 | 1.00 | 0.67 | 0.83 | 0.95 | 0.80 | 0.87 | 0.92 | 0.64 | 0.75 | 0.82 | - |

**S2 Table (part 2/2)**
