# Supplementary material for: To What Extent is Primate Second Molar Enamel Occlusal Morphology Shaped by the Enamel-Dentine Junction?
Source: PLoS One. 2015 Sep 25;10(9):e0138802. doi: 10.1371/journal.pone.0138802 (PMC4634312; doi:10.1371/journal.pone.0138802)
Supplement: S3 Table — 3DET, average 3D occlusal thickness (mm); 3DETSTD, average standardized 3D occlusal thickness; 3DOPCOES, 3D orientation patch count for the OES; 3DOPCEDJ, 3D orientation patch count for the EDJ. See S2 Table for specimen names. (DOCX) [file pone.0138802.s008.docx]

**S3 Table. Computed occlusal patch count (OES and EDJ) and enamel thickness variables for the primate molar sample.** 3DET, average 3D occlusal thickness (mm); 3DETSTD, average standardized 3D occlusal thickness; 3DOPCOES, 3D orientation patch count for the OES; 3DOPCEDJ, 3D orientation patch count for the EDJ. See S2 Table for specimen names.

|  | **3DET** | **3DET^STD^** | **3DOPC^OES^** | **3DOPC^EDJ^** |  |  | **3DET** | **3DET^STD^** | **3DOPC^OES^** | **3DOPC^EDJ^** |
| --- | --- | --- | --- | --- | --- | --- | --- | --- | --- | --- |
| **Hsap_#1** | 1.44 | 1.41 | 67 | 89 |  | **Lophat_#1** | 0.70 | 1.06 | 112 | 69 |
| **Hsap_#2** | 1.41 | 1.28 | 96 | 108 |  | **Lophalb_#1** | 0.79 | 1.10 | 85 | 74 |
| **Hsap_#3** | 1.49 | 1.51 | 86 | 82 |  | **Lophalb_#2** | 0.67 | 1.03 | 75 | 60 |
| **Hsap_#4** | 1.51 | 1.51 | 77 | 75 |  | **Lophalb_#3** | 0.79 | 1.09 | 82 | 69 |
| **Hsap_#5** | 1.44 | 1.48 | 85 | 89 |  | **Cercsp_#1** | 0.51 | 0.85 | 72 | 64 |
| **Hsap_#6** | 1.34 | 1.52 | 67 | 62 |  | **Cercsp_#2** | 0.62 | 1.03 | 73 | 72 |
| **Hsap_#7** | 1.32 | 1.34 | 70 | 106 |  | **Cerccamp_#1** | 0.49 | 0.74 | 75 | 56 |
| **Ppan_#1** | 0.89 | 0.93 | 173 | 125 |  | **Cerccamp_#2** | 0.54 | 0.92 | 64 | 84 |
| **Ppan_#2** | 0.71 | 0.84 | 202 | 171 |  | **Cercpog_#1** | 0.53 | 0.81 | 93 | 85 |
| **Ppan_#3** | 0.74 | 0.78 | 141 | 118 |  | **Cercpog_#2** | 0.57 | 0.98 | 77 | 82 |
| **Ppan_#4** | 0.82 | 0.94 | 151 | 108 |  | **Cerccep_#1** | 0.46 | 0.64 | 84 | 96 |
| **Ppan_#5** | 0.78 | 0.83 | 162 | 129 |  | **Cercnic_#1** | 0.45 | 0.84 | 63 | 90 |
| **Ppan_#6** | 0.86 | 0.97 | 115 | 152 |  | **Erythpat_#1** | 0.53 | 0.71 | 89 | 81 |
| **Ppan_#7** | 0.87 | 0.90 | 216 | 168 |  | **Papsp_#1** | 1.20 | 1.00 | 110 | 99 |
| **Ggor_#1** | 1.00 | 0.66 | 113 | 121 |  | **Papsp_#2** | 1.07 | 0.95 | 120 | 82 |
| **Ggor_#2** | 1.16 | 0.83 | 96 | 71 |  | **Papsp_#3** | 1.04 | 0.87 | 104 | 97 |
| **Ggor_#3** | 1.10 | 0.76 | 114 | 97 |  | **Procver_#1** | 0.33 | 0.58 | 103 | 86 |
| **Ggor_#4** | 0.91 | 0.67 | 88 | 117 |  | **Procver_#2** | 0.30 | 0.54 | 76 | 85 |
| **Ggor_#5** | 1.11 | 0.76 | 144 | 116 |  | **Procver_#3** | 0.33 | 0.61 | 73 | 74 |
| **Ggor_#6** | 1.23 | 0.76 | 190 | 150 |  | **Colpol_#1** | 0.45 | 0.69 | 96 | 75 |
| **Ggor_#7** | 1.10 | 0.79 | 120 | 88 |  | **Colpol_ #2** | 0.52 | 0.69 | 71 | 78 |
| **Ptro_#1** | 0.83 | 0.85 | 112 | 120 |  | **Colpol_ #3** | 0.44 | 0.64 | 97 | 97 |
| **Ptro_#2** | 0.81 | 0.83 | 177 | 114 |  | **Colpol_ #4** | 0.56 | 0.88 | 89 | 87 |
| **Ptro_#3** | 0.91 | 0.90 | 140 | 127 |  | **Colpol_ #5** | 0.51 | 0.72 | 121 | 107 |
| **Ptro_#4** | 0.72 | 0.66 | 291 | 167 |  | **Colbad_#1** | 0.48 | 0.70 | 91 | 70 |
| **Ptro_#5** | 0.72 | 0.75 | 158 | 160 |  | **Colbad_#2** | 0.54 | 0.75 | 94 | 76 |
| **Ptro_#6** | 1.04 | 1.06 | 175 | 103 |  | **Colguer_#1** | 0.52 | 0.67 | 117 | 74 |
| **Ptro_#7** | 0.80 | 0.85 | 191 | 176 |  | **Semenent_#1** | 0.55 | 0.81 | 83 | 71 |
| **Ptro_#8** | 0.85 | 0.86 | 212 | 184 |  | **Allousp_#1** | 0.33 | 0.62 | 125 | 106 |
| **Ptro_#9** | 0.78 | 0.81 | 183 | 147 |  | **Calljac_#1** | 0.12 | 0.68 | 62 | 57 |
| **Ptro_#10** | 0.91 | 0.82 | 237 | 135 |  | **Calljac_#2** | 0.12 | 0.68 | 51 | 60 |
| **Hyl_#1** | 0.53 | 0.83 | 66 | 93 |  | **Lagsp_#1** | 0.25 | 0.53 | 92 | 79 |
| **Hyl_#2** | 0.53 | 0.84 | 64 | 72 |  | **Lagsp_#2** | 0.29 | 0.61 | 77 | 83 |
| **Cerbsp_#1** | 0.68 | 0.96 | 86 | 63 |  | **Laglag_#1** | 0.46 | 0.86 | 115 | 77 |
| **Cerbsp_#2** | 0.82 | 1.01 | 77 | 88 |  | **Callcup_#1** | 0.24 | 0.69 | 175 | 141 |
| **Cerbtor_#1** | 0.74 | 0.91 | 102 | 79 |  | **Callcup_#2** | 0.21 | 0.62 | 148 | 125 |
| **Cerbtor_#2** | 0.74 | 0.90 | 102 | 79 |  | **Cebap_#1** | 0.36 | 0.73 | 95 | 88 |
| **Cerbgal_#1** | 0.69 | 0.94 | 127 | 74 |  | **Lemsp_#1** | 0.19 | 0.37 | 133 | 72 |

**S3 Table**
